# Endotoxemia by *Porphyromonas gingivalis* Injection Aggravates Non-alcoholic Fatty Liver Disease, Disrupts Glucose/Lipid Metabolism, and Alters Gut Microbiota in Mice

**DOI:** 10.3389/fmicb.2018.02470

**Published:** 2018-10-24

**Authors:** Naoki Sasaki, Sayaka Katagiri, Rina Komazaki, Kazuki Watanabe, Shogo Maekawa, Takahiko Shiba, Sayuri Udagawa, Yasuo Takeuchi, Anri Ohtsu, Takashi Kohda, Haruka Tohara, Naoyuki Miyasaka, Tomomitsu Hirota, Mayumi Tamari, Yuichi Izumi

**Affiliations:** ^1^Department of Periodontology, Graduate School of Medical and Dental Sciences, Tokyo Medical and Dental University, Tokyo, Japan; ^2^Department of Epigenetics, Medical Research Institute, Tokyo Medical and Dental University, Tokyo, Japan; ^3^Japan Agency for Medical Research and Development (AMED), Tokyo, Japan; ^4^Faculty of Life and Environmental Sciences, University of Yamanashi, Yamanashi, Japan; ^5^Gerodontology and Oral Rehabilitation, Department of Gerontology and Gerodontology, Graduate School of Medical and Dental Sciences, Tokyo Medical and Dental University, Tokyo, Japan; ^6^Department of Comprehensive Reproductive Medicine, Tokyo Medical and Dental University, Tokyo, Japan; ^7^Research Center for Medical Science, Core Research Facilities for Basic Science (Molecular Genetics), The Jikei University School of Medicine, Tokyo, Japan

**Keywords:** *Porphyromonas gingivalis*, endotoxemia, non-alcoholic fatty liver disease, periodontitis, gut microbiota

## Abstract

Many risk factors related to the development of non-alcoholic fatty liver disease (NAFLD) have been proposed, including the most well-known of diabetes and obesity as well as periodontitis. As periodontal pathogenic bacteria produce endotoxins, periodontal treatment can result in endotoxemia. The aim of this study was to investigate the effects of intravenous, sonicated *Porphyromonas gingivalis* (*Pg*) injection on glucose/lipid metabolism, liver steatosis, and gut microbiota in mice. Endotoxemia was induced in C57BL/6J mice (8 weeks old) by intravenous injection of sonicated *Pg*; *Pg* was deactivated but its endotoxin remained. The mice were fed a high-fat diet and administered sonicated *Pg* (HFPg) or saline (HFco) injections for 12 weeks. Liver steatosis, glucose metabolism, and gene expression in the liver were evaluated. 16S rRNA gene sequencing with metagenome prediction was performed on the gut microbiota. Compared to HFco mice, HFPg mice exhibited impaired glucose tolerance and insulin resistance along with increased liver steatosis. Liver microarray analysis demonstrated that 1278 genes were differentially expressed between HFco and HFPg mice. Gene set enrichment analysis showed that fatty acid metabolism, hypoxia, and TNFα signaling via NFκB gene sets were enriched in HFPg mice. Although sonicated *Pg* did not directly reach the gut, it changed the gut microbiota and decreased bacterial diversity in HFPg mice. Metagenome prediction in the gut microbiota showed enriched citrate cycle and carbon fixation pathways in prokaryotes. Overall, intravenous injection of sonicated *Pg* caused impaired glucose tolerance, insulin resistance, and liver steatosis in mice fed high-fat diets. Thus, blood infusion of *Pg* contributes to NAFLD and alters the gut microbiota.

## Introduction

Non-alcoholic fatty liver disease (NAFLD) is the hepatic manifestation of metabolic syndrome (Jensen et al., 2018). A multiple-hit hypothesis has been proposed in which multiple insults, including lipopolysaccharide (LPS), gut microbiota, nutritional factors, and genetic and epigenetic factors, act together to induce NAFLD (Buzzetti et al., 2016). NAFLD is strongly associated with type 2 diabetes, obesity, and insulin resistance (Malaguarnera et al., 2009; Leite et al., 2011; Sanyal, 2011); however, other risk factors for the aggravation of NAFLD have not been sufficiently clarified to date.

Periodontal disease is a chronic infectious disease triggered by periodontal bacteria in dental plaque, resulting in an inflammatory loss of bone and both the soft and hard tooth-supporting structures (Pihlstrom et al., 2005; Nassar et al., 2007). Periodontal bacteria, including *Porphyromonas gingivalis*, produce various virulence factors such as LPS, fimbriae, and enzymes, which can lead to inflammation in periodontal tissues (Kolenbrander et al., 2002).

The relationship between infection by periodontal bacteria and NAFLD has recently attracted substantial research attention (Yoneda et al., 2012; Komazaki et al., 2017). Yoneda et al. (2012) first reported that infection with *P. gingivalis* may be a risk factor for the development and progression of NAFLD. We also reported that swallowing of the periodontal pathogenic bacteria *Aggregatibacter actinomycetemcomitans* changes the gut microbiota and is a possible risk factor for NAFLD (Komazaki et al., 2017). In addition, in ligature-induced periodontitis, periodontal bacteria naturally accumulate around the teeth, and also increase liver steatosis in rats (Kuraji et al., 2016; Pessoa et al., 2018). Imajo et al. (2012) reported that the response to low-dose LPS was enhanced by liver steatosis-induced high-fat diet feeding, and additional low-dose LPS administration led to liver injury and severe hepatic fibrosis in mice.

The current strategy for periodontal treatment is to remove the periodontal bacteria around the teeth (Lindhe et al., 2015). However, there have been many reports of endotoxemia occurring after periodontal treatment accompanied by increased production of inflammatory cytokines (Ide et al., 2004; Forner et al., 2006; Tonetti et al., 2007). Clinically, ultrasonic scaling devices are applied for periodontal treatment to destroy and remove the microbial composition. However, there have been no studies evaluating whether endotoxemia itself from *P. gingivalis* affects NAFLD or glucose/lipid metabolism.

To resolve these issues, in the present study, we investigated the effect of intravenously injected sonicated *P. gingivalis* on glucose/lipid metabolism and liver steatosis. In addition, we evaluated the effects of a metabolic change from injected rather than swallowed sonicated *P. gingivalis* on gut microbiota.

## Materials and Methods

### Animals

C57BL/6J mice (8 weeks old; Sankyo Laboratory, Tokyo, Japan) were used in this study, and allowed free access to water and food throughout the experimental period. The mice were fed High-fat diet 32 (CLEA Japan, Inc., Tokyo, Japan), which contains 506.8 kcal/100 g (57.5% from fat, 19.7% from protein, and 22.8% from carbohydrate). High-fat diet 32 is composed of 24.5% milk casein, 5.0% albumin powder, 0.43% L-cystine, 15.88% powdered beef tallow, 20.0% safflower oil, 5.5% crystalline cellulose, 8.25% maltodextrin, 6.928% lactose, 6.75% sucrose, 1.4% American Institute of Nutrition (AIN)-93 vitamin mix, 5.0% AIN-93G mineral mix, 0.36% choline hydrogen, and 0.002% tertiary butyl hydroquinone. The mice were randomly divided into a control group (HFco, *n* = 10) and a group receiving intravenous injection of sonicated *P. gingivalis* twice a week (HFPg, *n* = 10) for 12 weeks. This experimental period was chosen because Imajo et al. (2012) reported that low-dose LPS strongly affected liver steatosis only in high-fat diet-fed mice, and not in normal chow diet-fed mice. We also previously reported enhanced lipid accumulation in the liver only in the high-fat diet-fed mice after 12 weeks, which was neither detected in normal chow diet-fed mice nor at 6 weeks (Komazaki et al., 2017). Yoneda et al. (2012) also reported that high-fat diet-fed mice intravenously injected with *P. gingivalis* showed a marked increase in body and liver weight after 12 weeks. A total of 10^8^ colony-forming units (CFU) of sonicated *P. gingivalis* suspended in 100 μl of physiological saline solution was given to the HFPg mice by intravenous injection. HFco mice were given saline only. An oral glucose tolerance test (GTT, *n* = 9) and insulin tolerance test (ITT, *n* = 9) were performed as previously described (Komazaki et al., 2017).

All protocols pertaining to animal use and euthanasia were reviewed and approved by the Animal Care Committee of the Experimental Animal Center at Tokyo Medical and Dental University (0170225A). The detailed protocol is described in Supplementary Information S1. The animal experiments were performed in accordance with the Guideline for the Care and Use of Laboratory Animals at Tokyo Medical and Dental University.

### Cultivation of *P. gingivalis*

*P. gingivalis* was cultivated as previously described (Udagawa et al., 2018). In brief, the bacteria were maintained on trypticase soy agar (Difco Laboratories, Detroit, MI, United States) supplemented with 10% defibrinated horse blood, hemin, and menadione at 37°C under anaerobic conditions. After 48 h of incubation, *P. gingivalis* was inoculated into trypticase soy broth under anaerobic conditions. The *P. gingivalis* suspension was cultured at 37°C under anaerobic conditions to the mid-log phase, and 10^9^ CFU/ml of the bacterial suspension was sonicated at 20 amplitude for 5 min on ice using a Qsonica Q700 sonicator (Waken Btech Co., Ltd., Kyoto, Japan). *P. gingivalis* itself was deactivated, but its endotoxins remained. Since the endotoxin concentration in the sonicated *P. gingivalis* suspension was 5.7 ± 1.2 pg/ml, HFPg mice were determined to be injected with 0.57 ± 0.12 pg LPS at each injection.

### *In vivo* Evaluation of Visceral and Subcutaneous Fat

Micro-computed tomography (CT) imaging was performed with a RmCT2 micro-CT unit (Rigaku Corporation, Tokyo, Japan) as previously described (*n* = 5) (Kina-Tanada et al., 2017; Komazaki et al., 2017). The CT images were visualized and analyzed using CTAtlas Metabolic Analysis (ver. 2.03) software (Rigaku Corporation). Body fat was then divided into visceral fat and subcutaneous fat along the ribs.

### RNA Preparation and Quantitative Polymerase Chain Reaction (PCR)

Extraction of total RNA from the liver and quantitative PCR were performed as previously described (*n* = 5) (Komazaki et al., 2017); *36b4* was used as the reference gene for normalization. PCR primers used in this study are listed in Supplementary Table S2.

### Liver Histological Analysis

Liver samples were collected from the left hepatic lobes, fixed with 4% paraformaldehyde in PBS at 4°C for 24 h, and placed in 10% sucrose in PBS at 4°C until the tissues sank (6–12 h, *n* = 5). The buffer was then replaced with 30% sucrose in PBS, in which the tissues were stored overnight at 4°C. The tissues were then embedded in Optimal Cutting Temperature compound (sakura finetek japan, Tokyo, Japan), and 10 μm sections were cut using a CM3050 S cryostat (Leica, Wetzlar, Germany). The tissue sections were then stained with Oil Red O and counterstained with hematoxylin. The areas that did not include large vessels were chosen for histological analysis. The adipocyte cross-sectional area was measured using Adobe Photoshop CC Software (Adobe Systems, San Jose, CA, United States).

### Glycogen Measurements

Aliquots of liver lysates were added to microcentrifuge tubes containing 37.5% KOH and heated at 70°C for 30 min. Subsequently, 25% Na_2_SO_4_ was added and heated at 70°C for 15 min (*n* = 5). Glycogen was precipitated by adding 100% ethanol and the tubes were stored at -20°C overnight. The tubes were centrifuged, decanted, and stored at -20°C overnight again. The precipitates were hydrolyzed by heating at 95°C for 2 h in 2 N HCl. After being neutralized by 2 N NaOH, the supernatants were added to 1 M Tris and used for measurement of glucose. Glycogen content was expressed as glucose units in the liver (Toyoda et al., 2011; An et al., 2014). A glucose solution (Sigma, Kanagawa, Japan) was used as a standard. Hexokinase (Sigma) was added and its optical density (OD) value was measured at 340 nm (VMax microplate reader; Molecular Devices, San Jose, CA, United States).

### Triglyceride Measurements

Triglyceride was measured as previously described (Le Marchand et al., 1973; Fujii et al., 2008). In brief, aliquots of liver lysates were added to microcentrifuge tubes containing 37.5% KOH and heated at 70°C for 30 min, and then 100% ethanol was added and the tubes were placed at 55°C overnight (*n* = 5). Subsequently, 50% ethanol was added and the tubes were centrifuged. The supernatants were separated, treated with MgCl_2_, left on ice for 10 min, and then centrifuged again. The supernatants and a glycerol standard solution (Sigma) were placed in a 96-well plate, and free glycerol regent (Sigma) was added before OD measurement at 540 nm.

### Plasma Insulin Levels

Commercially available kits were used to determine the plasma levels of insulin (Ultra Sensitive Mouse Insulin ELISA Kit, Morinaga Institute of Biological Science, Inc., Kanagawa, Japan) (*n* = 5) according to the manufacturer’s protocols.

### Plasma and Bacterial Endotoxin Levels

Wako ES-test kit (Wako Pure Chemical Industries, Ltd., Osaka, Japan) was used for measurement of the endotoxin concentration in plasma and sonicated *P. gingivalis* suspension.

### Microarray and Data Analysis

The Agilent Low Input Quick Amp Labeling kit v2 (Agilent Technologies, Santa Clara, CA, United States) was used to generate cRNA with a sample input of 200 ng total RNA for single-color microarray (Cy3) analysis (*n* = 4). The cRNA was then hybridized onto an Agilent SurePrint G3 Unrestricted Gene Expression 8 × 60 K Microarray (Agilent). Fluorescence signals from the hybridized microarrays were detected using the Agilent Microarray Scanner System (Agilent). Raw microarray data were extracted using Feature Extraction Software (ver. 11.0.1.1; Agilent).

### 16S rRNA Gene Sequencing and Illumina Sequence Data Processing

DNA extraction from mouse feces (*n* = 4), purification, and generation of the multiplexed amplicon library (16S rDNA V3-V4 region) were performed as previously described (Komazaki et al., 2017). Paired-end sequences (250 bp) were produced by the Illumina Miseq platform (Illumina, Inc., San Diego, CA, United States). The sequence data are available in the DNA Data Bank of Japan (accession number: DRA006691). Low-quality sequences, pyrosequencing errors, noise, and chimeras were removed. We then clustered the preprocessed reads into operational taxonomic units (OTUs) at 97% identity using the CD-HIT-OTU pipeline^1^ (ver.0.01) (Li et al., 2012).

### Taxonomic Assignment and Metagenome Prediction

Operational taxonomic units were processed and analyzed with the Quantitative Insights into Microbial Ecology software (QIIME, ver. 1.8) (Caporaso et al., 2010). Taxonomic classification of the sequences at the phylum, family, and genus levels was carried out using the RDP classifier (ver. 2.2) with default parameters against the GreenGenes database (ver. :gg_13_8). Taxonomic assignment was refined at the species level based on the 16S rRNA database (DDBJ, as of March 9, 2018) using BLASTN. The PICRUSt (ver. 1.0.0) bioinformatics software package (Langille et al., 2013) was used to generate metabolic predictions based on closed OTUs. Randomly resampling the sequences to 10,000 reads per sample was performed to normalize the samples using the Seqtk application^2^. The analyzed OTUs were normalized to the 16S rRNA copy number. Functional composition of the data was predicted based on the Kyoto Encyclopedia of Genes and Genomes (KEGG) database (Kanehisa and Goto, 2000). Dendrograms with heatmaps were visualized using R (ver. 3.3.2). Dissimilarity values (1 – Pearson correlation) were clustered using average linkage methods.

### Statistical Analysis

In the animal experiments, Student’s *t*-test was applied to compare two groups using SPSS 22.0 software (SPSS Inc., Chicago, IL, United States); *P* < 0.05 was considered statistically significant. The Shapiro-Wilks test was performed to verify the data distribution, demonstrating that a parametric test was suitable for data analysis.

Microarray data were 75% tile-normalized and log_2_-transformed (Shippy et al., 2006) using R (ver. 3.3.2). The Limma Bioconductor package (ver. 3.30.4) (Ritchie et al., 2015) was used to identify differentially expressed genes (DEGs). Benjamin and Hochberg’s false discovery rate (FDR) was applied for multiple testing. DEGs were defined according to FDR < 0.05 and |fold-change| > 2.0. Overrepresentation enrichment analyses for DEGs were performed with the WEB-based Gene SeT AnaLysis Toolkit^3^ (Wang et al., 2013) and the Database for Annotation, Visualization and Integrated Discovery (DAVID^4^) using the Gene Ontology (GO) and KEGG pathway databases. Gene set enrichment analysis (Subramanian et al., 2005) (GSEA^5^) was carried out with hallmark gene sets (Liberzon et al., 2015).

## Results

### Sonicated *P. gingivalis* Injection Increased Body Weight, and Impaired Glucose Tolerance and Insulin Resistance

**FIGURE 1:**
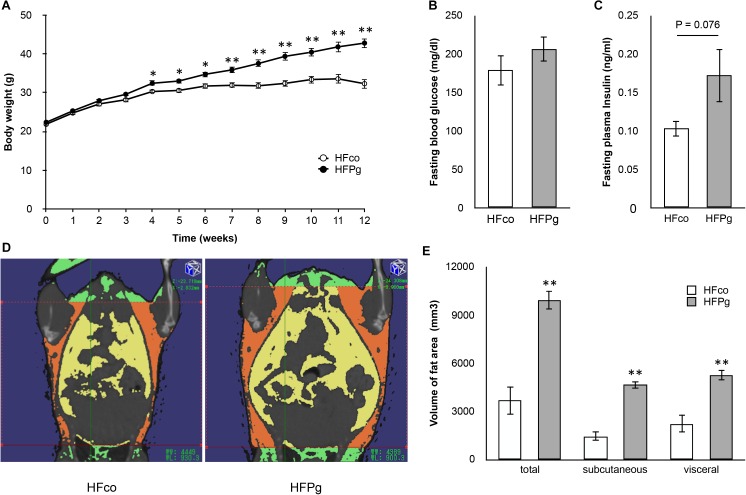
Comparison of body weight, fasting glucose/insulin concentration and body fat between HFco and HFPg mice. **(A)** Body weight **(B)** fasting blood glucose at 12 weeks **(C)** fasting plasma insulin at 12 weeks **(D)** Photographs of Micro-CT imaging. Yellow region represents visceral fat area and orange region represents subcutaneous fat area; **(E)** The volume of total fat area, subcutaneous fat area, and visceral fat area evaluated by Micro-CT imaging at 12 weeks (*n* = 5). ^∗^*P* < 0.05, ^∗∗^*P* < 0.01 HFco vs. HFPg (unpaired *t*-test).

The body weight of the HFPg mice was significantly increased 4 weeks after injection of sonicated *P. gingivalis* compared to that of the HFco mice. The differences in body weight were increased throughout the experimental periods (Figure 1A). Although there was no significant difference between fasting plasma glucose levels between the HFco and HFPg mice (Figure 1B), the HFPg mice tended (*P* = 0.076) to exhibit increased fasting plasma insulin 12 weeks after injection of sonicated *P. gingivalis* (Figure 1C). Three-dimensional micro-CT analysis showed that total body fat, subcutaneous fat, and visceral fat volumes were significantly higher in HFPg mice than in HFco mice at 12 weeks (Figures 1D,E). Endotoxin was not detected in the plasma collected from both groups of mice after 3 days of sonicated *P. gingivalis* or saline injection.

**FIGURE 2:**
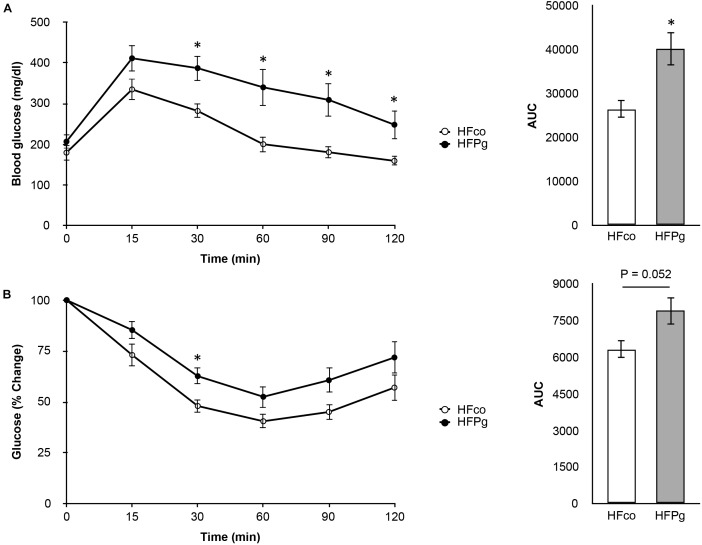
Comparison of glucose tolerance and insulin resistance between HFco and HFPg mice. **(A)** OGTT (1 g/kg) and **(B)** ITT (1.5 U/kg) performed 6 h fasting at 12 weeks (*n* = 9).^∗^*P* < 0.05 HFco vs. HFPg (unpaired *t*-test).

To determine whether administration of sonicated *P. gingivalis* induced impaired glucose tolerance and insulin resistance, we carried out a GTT (Figure 2A) and ITT (Figure 2B) at 12 weeks. Injection of sonicated *P. gingivalis* caused impaired glucose tolerance and insulin resistance.

### Sonicated *P. gingivalis* Injection Increased Liver Steatosis and Changed Expression Levels of Genes Related to Lipid and Glucose Metabolism in the Liver

**FIGURE 3:**
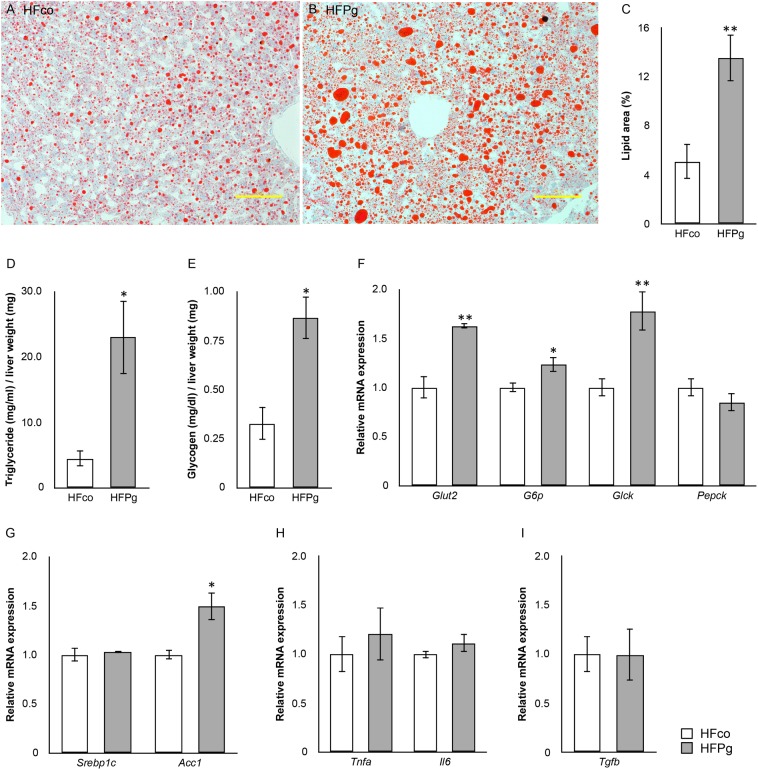
evaluation of liver steatosis. Oil red o staining of liver tissue from **(A)** HFco, **(B)** HFg mice (row magnification × 200, yellow bar = 100 μm), and **(C)** lipid area (%). **(D)** Triglyceride and **(E)** glycogen in the liver of HFco and HFPg mice at 12 weeks (*n* = 5). **(F)** *Glut2*, *G6p*, *Glck*, and *Pepck*; **(G)** *Srebp1c* and *Acc1*; **(H)** *Tnfa* and *Ilb*; **(I)** *Tgfb* expressions in HFco and HFPg mice at 12 weeks (*n* = 5).^∗^*P* < 0.05, ^∗∗^*P* < 0.01 (unpaired *t*-test).

Histological analysis showed marked lipid accumulation in HFPg (Figure 3B) compared to that in HFco (Figure 3A) mice after 12 weeks. The results showed that the total area of lipid droplets was significantly increased in HFPg mice compared to that in HFco mice (*P* < 0.01) (Figure 3C). Hepatic triglyceride (Figure 3D) and glycogen (Figure 3E) levels were significantly increased after 6 h of fasting at 12 weeks in HFPg mice compared to those in HFco mice.

Intravenous administration of sonicated *P. gingivalis* led to increased mRNA expression levels of glucose transporter 2 (*Glut2*), glucose-6-phosphate (*G6p*), glucokinase (*Glck*), and acetyl-CoA carboxylase (*Acc1*) in the liver of HFPg mice (Figures 3F,G). mRNA expression levels of tumor necrosis factor-alpha (*Tnfa*), interleukin-6 (*Il6*), and transforming growth factor-beta (*Tgfb*) in the liver did not differ significantly between HFco and HFPg mice at 12 weeks (Figures 3H,I).

### Liver Microarray Analysis After Sonicated *P. gingivalis* or Saline Injection

To identify changes in gene expression in the liver following injection of sonicated *P. gingivalis*, a comprehensive microarray analysis was carried out.

**FIGURE 4:**
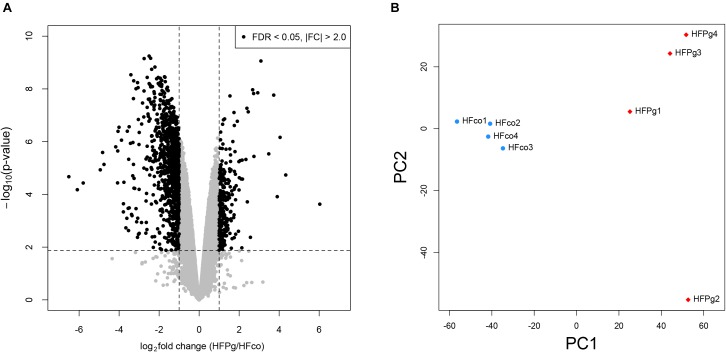
Microarray analysis in the liver between HFco and HFPg mice (*n* = 4). **(A)** Volcano plots, **(B)** PCA analysis.

**FIGURE 5:**
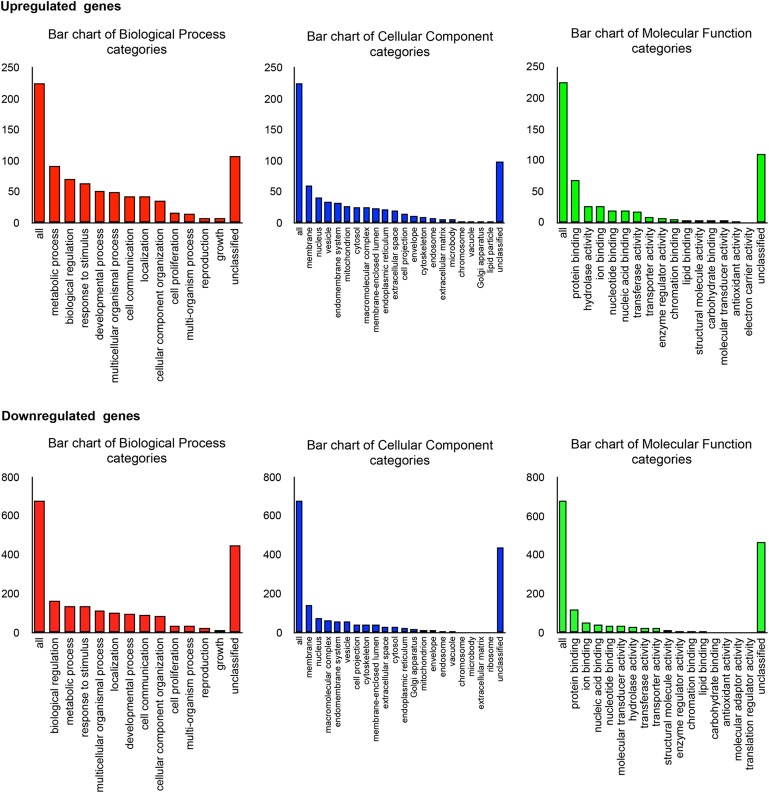
Microarray analysis in the liver between HFco and HFPg mice (*n* = 4). Gene Ontology in DEGs.

As shown in Figure 4A, a total of 1278 DEGs were identified, 258 of which were upregulated. Gene expression patterns in the livers of HFco and HFPg mice differed substantially (Figure 4B). All DEGs and corresponding GO terms are listed in Supplementary Table S3. GO slim overviewed the ontology content in upregulated and downregulated DEGs, respectively (Figure 5). Notably, 41% of upregulated DEGs with GO terms were classified as “metabolic process” in the biological process category, although only 24% of the downregulated DEGs were classified as “metabolic process”.

**FIGURE 6:**
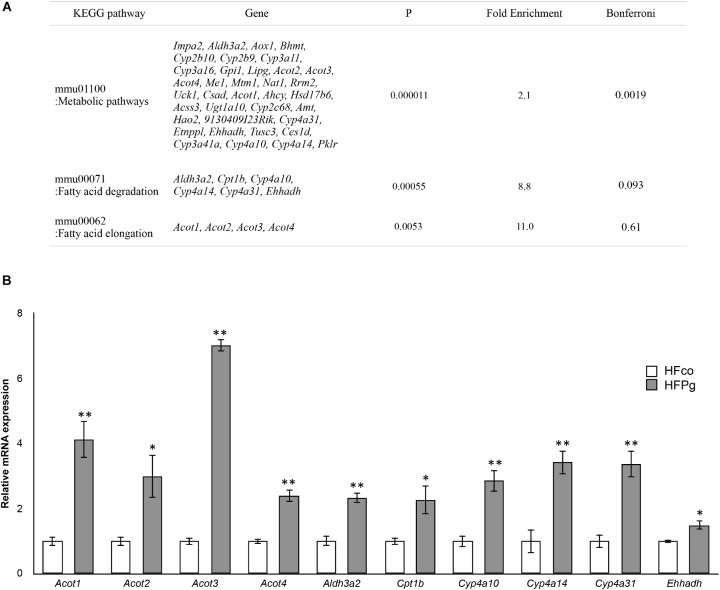
Microarray analysis in the liver between HFco and HFPg mice. **(A)** KEGG pathways in the upregulated DEGs (*n* = 4), **(B)** *Acot1*, *Acot2*, *Acot3*, *Acot4*, *Aldh3a2*, *Cpt1b*, *Cyp4a10*, *Cyp4a14*, *Cyp4a31*, and *Ehhadh* expressions in the HFco and HFPg mice at 12 weeks (*n* = 5) ^∗^*P* < 0.05, ^∗∗^*P* < 0.01 (unpaired *t*-test).

Pathway analysis showed that the metabolic pathway was significantly enriched in upregulated DEGs. In addition, fatty acid degradation and fatty acid elongation were enriched in upregulated DEGs (Figure 6A). The upregulation of genes related to fatty acid degradation and fatty acid elongation in the KEGG pathway were validated based on quantification of mRNA expression levels with qPCR. Specifically, *Acot1*, *Acot2*, *Acot3*, *Acot4*, *Aldh3a2*, *Cpt1b*, *Cyp4a10*, *Cyp4a14*, *Cyp4a31*, and *Ehhadh* expression levels were significantly increased after administration of sonicated *P. gingivalis* (Figure 6B). There was no significantly enriched pathway in downregulated DEGs.

**FIGURE 7:**
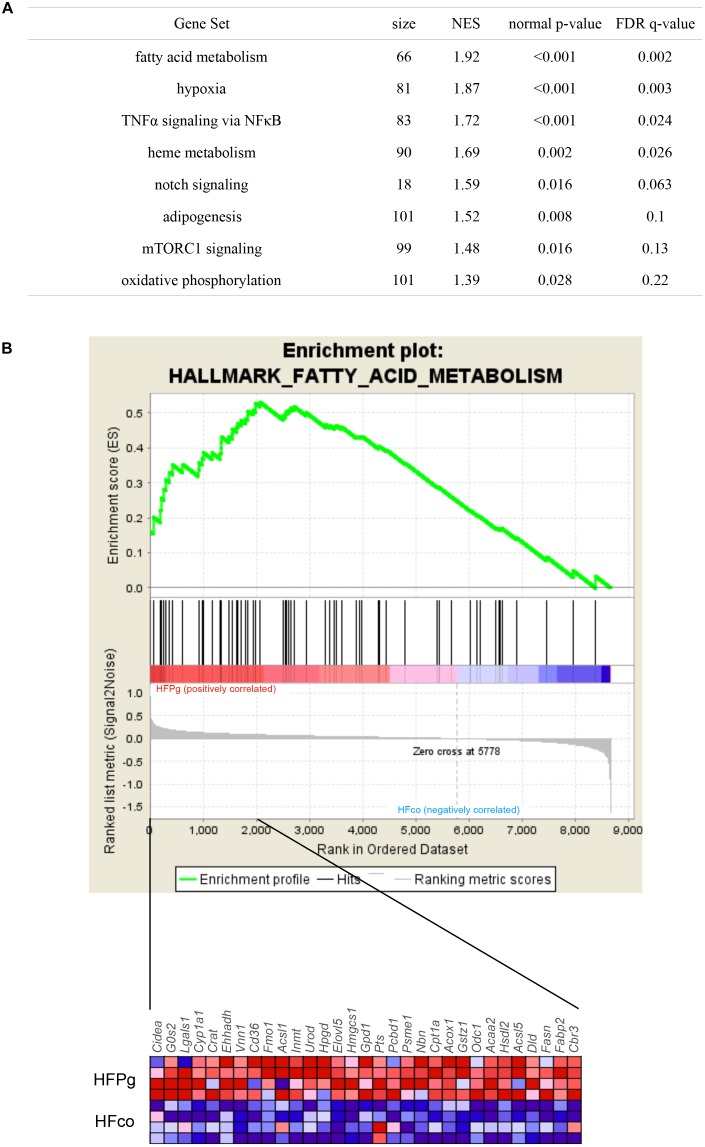
GSEA with hallmark gene sets enriched in HFPg mice compared to HFco mice (*n* = 4). **(A)** Gene sets showing FDR *q* < 0.25. NES: normalized enrichment score. **(B)** Gene set related to fatty acid metabolism. A heatmap provided illustrating gene expression levels for each gene in the core enrichment subset (blue: low, red: high).

GSEA was performed using hallmark gene sets to evaluate differences in mRNA expression levels in the livers of mice in the HFco and HFPg groups. The gene sets in Figure 7A show those with a FDR *q* < 0.25. Although no downregulated gene set with a FDR *q* < 0.25 was found in HFPg mice, several gene sets, including sets related to hypoxia, TNFα signaling via NFκB, and adipogenesis, were upregulated in HFPg mice. Notably, a gene set related to fatty acid metabolism was strongly upregulated (normalized enrichment score = 1.92, *q* = 0.002) in HFPg mice (Figure 7B).

### Evaluation of Gut Microbiome Composition Based on 16S rRNA Gene Sequences

**FIGURE 8:**
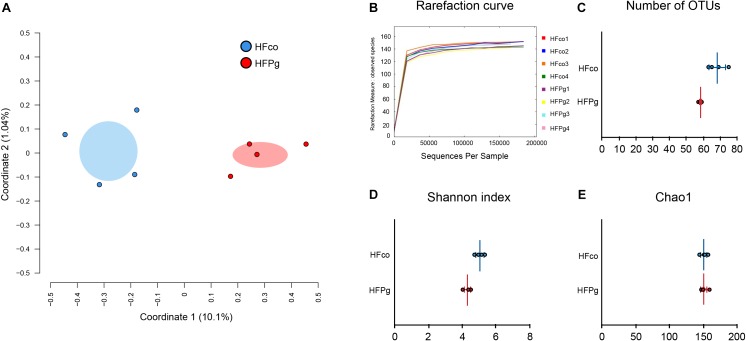
Evaluation of gut microbiome compositions based on 16S rRNA gene sequences between HFco and HFPg mice (*n* = 4). **(A)** PCoA analysis, **(B)** rarefaction curve, **(C)** number of OTUs, **(D)** Shannon index, **(E)** Chao1 index between HFco and HFPg mice.

A total of 4,667,794 sequence reads were generated corresponding to an average of 583,474 (range, 519,954–624,076) reads per sample. Principal coordinate analysis (PCoA) revealed different microbiome compositions in the HFco and HFPg groups. The first principal coordinate (PC1) indicated that 10.1% of the total variance was due to the difference between the two groups (Figure 8A). Rarefaction curves indicated that a sufficient number of reads were obtained for 16S rRNA gene analysis (Figure 8B). In addition, the number of OTUs (*P* < 0.05) (Figure 8C) and Shannon index (*P* < 0.01) (Figure 8D) differed significantly in the HFco and HFPg groups, although the Chao1 index of both groups was comparable (Figure 8E).

**FIGURE 9:**
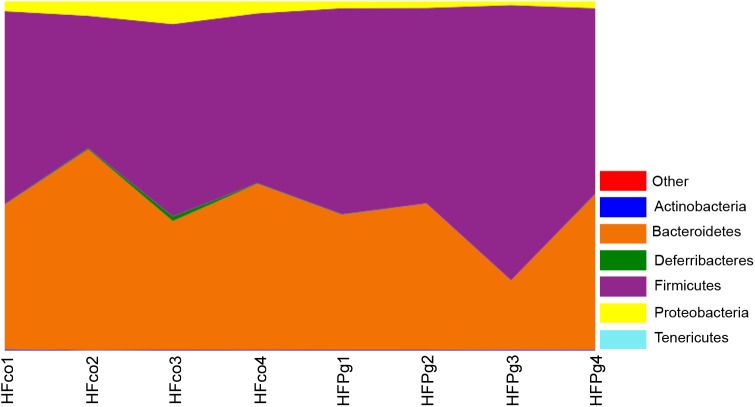
Evaluation of gut microbiome compositions based on 16S rRNA gene sequences between HFco and HFPg mice at a Phylum level (*n* = 4) (unpaired *t*-test with FDR).

**FIGURE 10:**
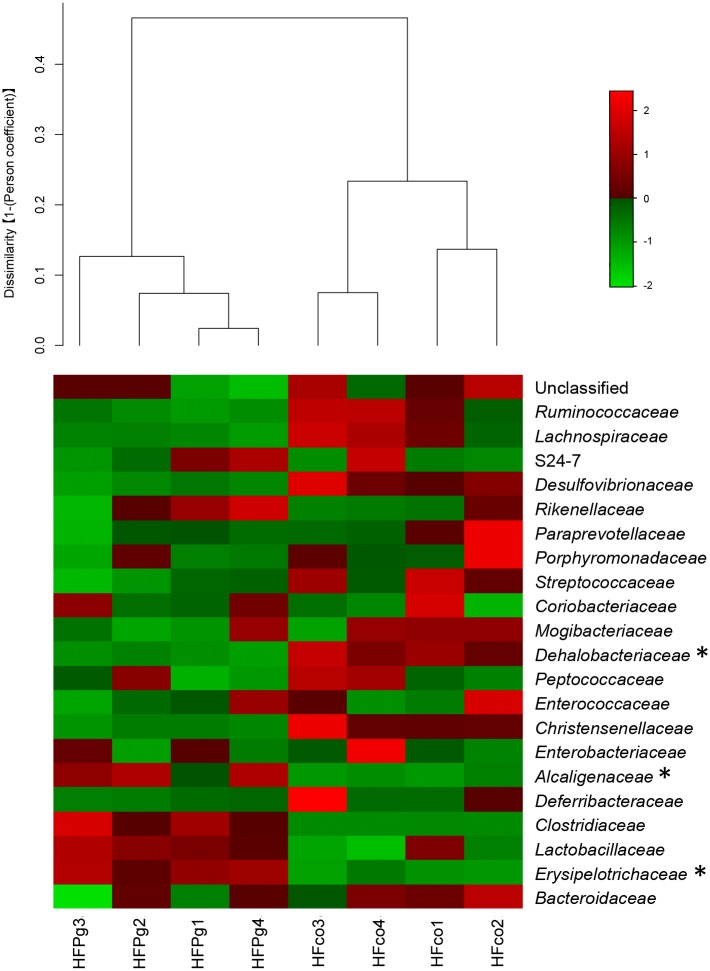
Evaluation of gut microbiome compositions based on 16S rRNA gene sequences between HFco and HFPg mice at a Family level (*n* = 4). Dendrogram and heatmap was constructed based on read abundance. ^∗^adjusting *P* < 0.05 between HFco and HFPg mice (unpaired *t*-test with FDR).

**FIGURE 11:**
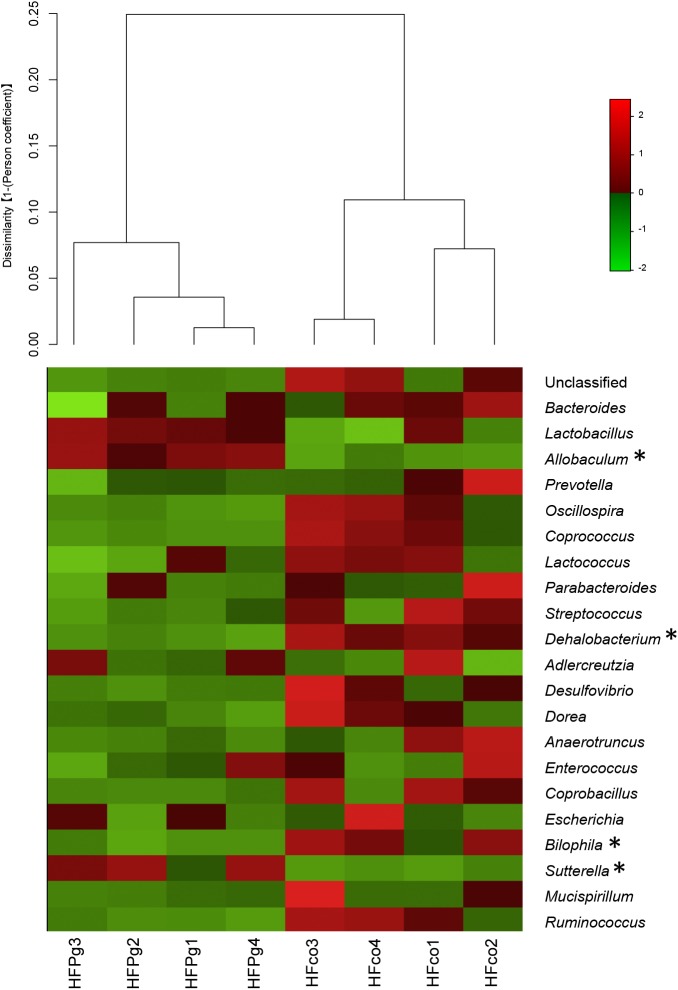
Evaluation of gut microbiome compositions based on 16S rRNA gene sequences between HFco and HFPg mice at a Genus level (*n* = 4). Dendrogram and heatmap constructed based on read abundance. ^∗^adjusting *P* < 0.05 between HFco and HFPg mice (unpaired *t*-test with FDR).

**FIGURE 12:**
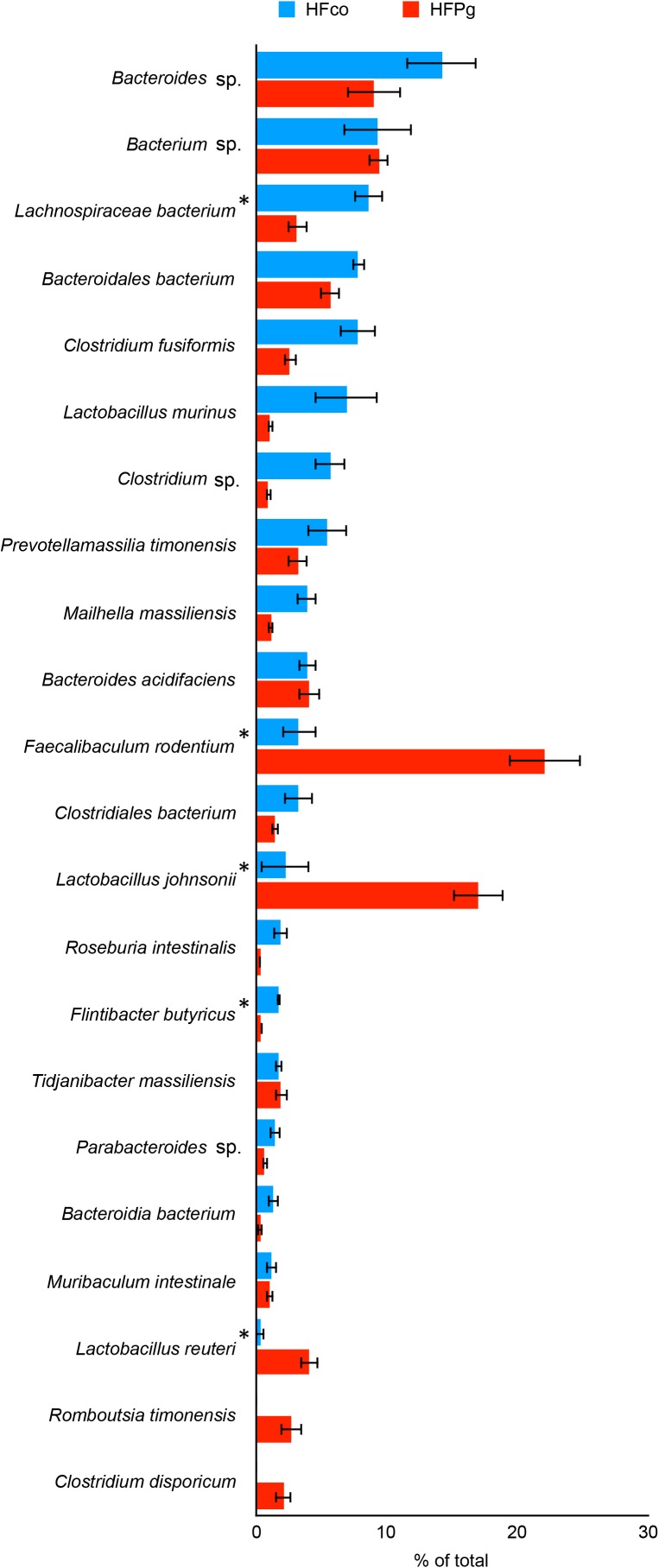
Evaluation of gut microbiome compositions based on 16S rRNA gene sequences between HFco and HFPg mice (*n* = 4). Distributions of the species between HFco and HFPg mice (>1.0% relative abundance). The species name or 16S ribosomal RNA database ID in DDBJ is shown. ^∗^adjusting *P* < 0.05 between HFco and HFPg mice (unpaired *t*-test with FDR).

Although no significant differences between gut microbiota composition were observed in HFco and HFPg mice at the phylum level (Figure 9), the phyla Tenericutes (*P* = 0.03, *q* = 0.11) and Proteobacteria (*P* = 0.03, *q* = 0.18) tended to decrease after sonicated *P. gingivalis* injection. Notably, Tenericutes was unrepresented in three of four HFPg mice. In addition, significant increases of the families *Alcaligenaceae* and *Erysipelotrichaceae*, and decrease of the family *Dehalobacteriaceae* were observed in HFPg mice. By contrast, the family *Ruminococcaceae* tended to be decreased (*P* = 0.012, FDR *q* = 0.065) in HFPg mice compared to HFco mice (Figure 10). The genera *Bilophila* and *Dehalobacterium* were significantly underrepresented in HFPg compared to HFco mice, whereas the genera *Sutterella* and *Allobaculum* were significantly overrepresented in HFPg compared to HFco mice (Figure 11). The most abundant species (>1.0%) in HFco and HFPg mice are presented in Figure 12. *Faecalibaculum rodentium*, *Lactobacillus johnsonii*, and *Lactobacillus reuteri* were significantly overrepresented in HFPg mice compared to HFco mice. All OTU species assignments are listed in Supplementary Table S4.

### Metagenome Prediction of the Gut Microbiome

**FIGURE 13:**
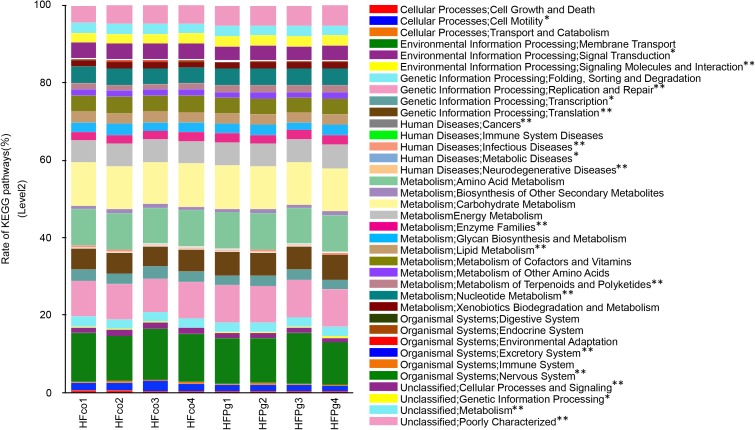
Metagenome prediction of level-2 subsystem between HFco and HFPg mice (*n* = 4). ^∗^adjusting *P* < 0.05 between HFco and HFPg mice (unpaired *t*-test with FDR).

PICRUSt analysis provided predictions of the relative abundance of gene function in the gut microbiome. In HFPg mice, functional composition at level 2 significantly increased with respect to cell motility, transcription, lipid metabolism, cellular processes and signaling, signal transduction, metabolism, and neurodegenerative diseases. However, the excretory system, metabolic diseases, nervous system, cancers, infectious diseases, signaling molecules and interaction, genetic information processing, metabolism of terpenoids and polyketides, enzyme families, nucleotide metabolism, replication and repair, and translation were decreased in HFPg mice (Figure 13).

**FIGURE 14:**
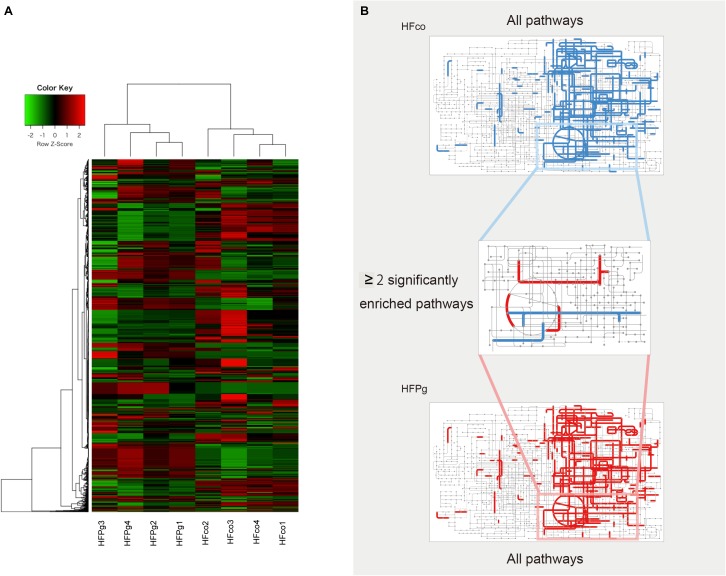
Metagenome prediction between HFco and HFPg mice. (*n* = 4) **(A)** Dendrogram and heatmap constructed based on metagenome prediction. **(B)** Predicted KEGG pathways present in any of samples for HFco (upper figure) and HFPg (lower figure). Middle figure shows significantly enriched pathway. Blue, HFco. Red, HFPg.

As shown in Figure 14A, metagenome predictions in HFco and HFPg mice were drastically different, and 1386 functional profiles were predicted to exhibit significant differences between HFco and HFPg mice (Supplementary Table S5). Interestingly, the citrate cycle, pyruvate metabolism, carbon fixation pathways in prokaryotes, and sulfur metabolism were significantly increased, whereas nicotinate and nicotinamide metabolism, chlorocyclohexane and chlorobenzene degradation, and chloroalkane and chloroalkene degradation were decreased in HFPg compared to HFco mice (Figure 14B).

## Discussion

Although we performed intravenous injection of deactivated *P. gingivalis*, remarkable liver steatosis was observed in the HFPg mice. Surprisingly, the gut microbiota was also changed by injection of deactivated *P. gingivalis*. Moreover, sonicated *P. gingivalis* injection caused insulin resistance and impaired glucose tolerance. These results are similar to those of previous studies using live *P. gingivalis* gavage (Arimatsu et al., 2014). Intravenous injection of live *P. gingivalis* increased the body weight and liver steatosis in mice (Yoneda et al., 2012). Our results also showed increased body weight along with increased total body fat, subcutaneous fat, and visceral fat volumes. However, we could not detect LPS from the plasma of *P. gingivalis* injected mice, which we suspect is due to the fact that the LPS injected in the blood immediately binds to LPS-binding protein (Horwitz et al., 1995).

In the liver, steatosis occurred with elevation of triglycerides and glycogen in HFPg mice. The liver possesses an insulin-independent facilitative glucose transport system based mostly on GLUT2. GLUT2 shows an apparent affinity constant of 17 mM and a high transport rate, allowing for remarkably rapid equilibration of the glucose concentration across the hepatocyte plasma membrane (Williams et al., 1968). *Glck*, the gene encoding glucokinase, which is the first enzyme in the pathway, increases the concentration of glucose 6-phosphate, which in turn regulates the phosphorylation state of downstream enzymes by acting synergistically with other allosteric effectors (Agius, 2008). Increased levels of *Glck* and *Glut2* were observed to depend on hyperglycemia, which could explain the increased glycogen levels observed in the HFPg mice. *Acc1* levels also increased in the livers of the HFPg mice in accordance with the increase in triglycerides. Several studies have reported that upregulation of ACC1 expression likely promotes lipogenesis to meet the needs for rapid growth and proliferation (Yahagi et al., 2005; Swinnen et al., 2006).

Although endotoxemia was induced in the HFPg mice, no increases in *Tnfa* or *Il6* levels were observed. In addition, no significant difference between *Tgfb* levels was observed in HFco and HFPg mice. This result differed from a previous study demonstrating that liver fibrosis was accelerated after *P. gingivalis* infection (Nakahara et al., 2018). We suppose that the virulence in our model is weaker since we injected sonicated *P. gingivalis* (ATCC 33277) twice a week, in contrast to the previous study in which high fat fed mice were continuously infected with live *P. gingivalis* (W83) by the dental pulp chamber model. In addition, *P. gingivalis* strains are classified into virulent (W83) and less-virulent (ATCC 33277) strains, and extensive genomic rearrangements were observed between the strains (Naito et al., 2008). We presume that the manner of *P. gingivalis* administration and the difference in the strains accounted for the different results obtained for liver fibrosis. Furthermore, microarray analysis was carried out to evaluate gene expression patterns in the liver exhaustively. The PCA plots varied widely in HFPg mice, but were substantially different between HFco and HFPg mice. We consider that this variation in HFPg mice is owing to the different susceptibility or reaction to sonicated *P. gingivalis* in each mouse. Upregulated DEGs showed higher GO term proportions for “metabolic process” in the biological process category. In addition, KEGG pathway analysis in upregulated DEGs showed enrichment of metabolic pathways and fatty acid elongation. The upregulation of genes related to fatty acid degradation and fatty acid elongation in KEGG pathway were validated based on detection of mRNA expression levels. These genes are also listed in metabolic pathway. Especially, *Acot1* and *Acot3* levels were drastically increased after *P. gingivalis* administration. These results demonstrated that fatty acid elongation was strongly enhanced, and fatty acid degradation was slightly increased to reduce liver steatosis. Acyl-CoA thioesterase 1 (ACOT1) regulates PPARα and fasting hepatic fatty acid metabolism by balancing oxidative flux and capacity (Franklin et al., 2017). In GSEA, eight of the hallmark gene sets showed *q* < 0.25. In accordance with the KEGG pathway analysis of the upregulated DEGs, the fatty acid metabolism and adipogenesis gene sets were enriched in the liver of HFPg mice. Interestingly, TNFα signaling via NFκB and hypoxia gene sets were upregulated in HFPg mice. Hypoxia-inducible factor 2α drives non-alcoholic fatty liver progression by triggering the hepatocyte release of histidine-rich glycoproteins (Morello et al., 2017). NFκB plays an essential role in inflammation and is related to the development of obesity-induced insulin resistance, metabolic syndrome, and NAFLD. TLR4 is an LPS receptor, which plays a vital role in innate immunity. Stimulation of TLR4 promotes pro-inflammatory signaling through induction of cytokine production, causing activation of the NFκB pathway (Zuany-Amorim et al., 2002). In our results, *Tnfα* levels were not significantly increased in the livers of HFPg mice compared to those of HFco mice. This suggests that LPS from sonicated *P. gingivalis* may have induced activation of NFκB.

Gut microbiota have been reported to change after oral administration of periodontal bacteria (Arimatsu et al., 2014; Komazaki et al., 2017). Surprisingly, in the present model, sonicated *P. gingivalis* did not directly reach the gut; however, the gut microbiota composition was still altered by intravenous injection of inactivated *P. gingivalis*. Previous studies demonstrated changes in the gut microbiota within 6 months of liver transplantation, with improved diversity, increased autochthonous taxa, and reduction of potentially pathogenic taxa (Bajaj et al., 2017, 2018). These reports might support our results that gut microbiota are altered after changes of the liver phenotype. Notably, although the PCoA demonstrated clear separation in the microbial diversity in HFco and HFPg mice, coordinate 1 indicated that only 10.1% of the total variance was due to differences between the groups. In addition, although the number of OTUs and Shannon index were decreased in HFPg mice, no significant difference was observed in the Chao1 index. These results suggest that intravenous injection of inactivated *P. gingivalis* caused significant but nevertheless slight changes to the gut microbiota of HFPg mice.

Some previous studies have demonstrated associations between the significant taxa identified in the present study and metabolic health. Lim et al. (2017) discovered the higher diversity and enrichment of members from the phylum Tenericutes in healthy subjects compared to those with metabolic syndrome. Daniel et al. (2014) reported that a high-fat diet changed the diversity of dominant gut bacteria and decreased the proportion of the family *Ruminococcaceae*. *Erysipelotrichaceae* in the gut microbiota was also reported to be enriched in NAFLD patients compared to a healthy subject group (Shen et al., 2017). Furthermore, the proportion of gut *Erysipelotrichaceae* was reported to be increased in mice fed a high-fat high-sucrose diet compared to that in mice fed a normal chow diet (Kishida et al., 2017). The intestinal microbiota of rats fed restrictive high-sugar diets reportedly has an increased abundance of Bacteroidetes and *Sutterella* bacteria with an accompanying increase of body weight, visceral fat, insulin resistance, and liver triglycerides (Liu et al., 2016). *Sutterella* spp. were found to be increased in prediabetic subjects compared to individuals with normal glucose regulation, although no significant association was observed between the abundance of *Sutterella* spp. and clinical biomarkers (Allin et al., 2018). The genus *Allobaculum* has been identified as the most active glucose utilizer and has been shown to yield mainly lactate and butyrate during glucose metabolism (Greetham et al., 2004). In addition, *Allobaculum* spp. abundance has been positively correlated with intestinal inflammation and increased gut permeability due to decreased expression levels of tight junction proteins (Lee et al., 2015). Thus, increases in these genera due to endotoxemia from *P. gingivalis* may further affect liver steatosis. *Lactobacillus reuteri* treatment reportedly increased insulin sensitivity in patients with type 2 diabetes undergoing insulin therapy (Mobini et al., 2017). In addition, administration of *Lactobacillus johnsonii* isolated from BioBreeding diabetes-resistant rats delayed or prevented the onset of type 1 diabetes in BioBreeding diabetes-prone rats (Valladares et al., 2010). In our model, the HFPg mice showed increased *L. reuteri* and *L. johnsonii* abundance compared to those in HFco mice, which might reflect a protection against the hyperglycemia caused by endotoxemia.

Metagenome prediction in the gut microbiota showed significant increases in the citrate cycle, pyruvate metabolism, carbon fixation pathways in prokaryotes, and sulfur metabolism. These enrichments might be explained by the increased abundance of anaerobic bacteria in the gut microbiota of HFPg mice.

**FIGURE 15:**
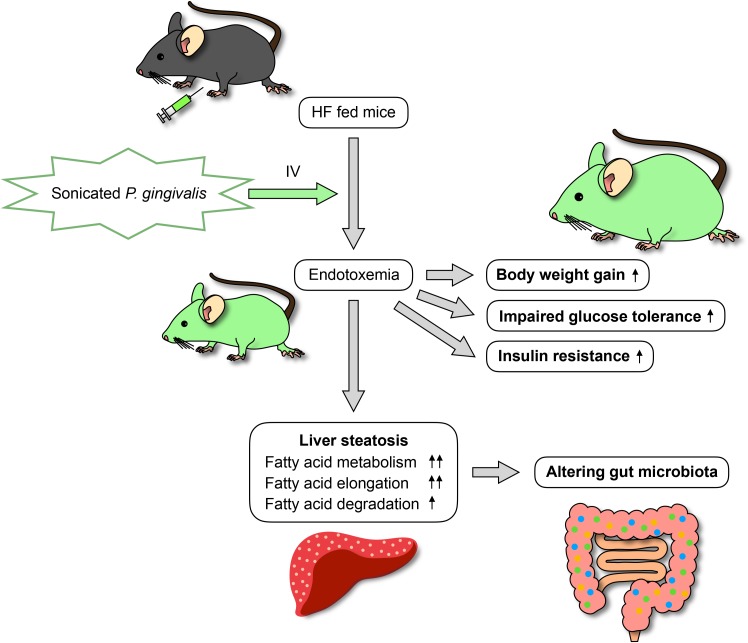
A mechanistic model summarizing the findings of the study.

In conclusion, endotoxemia from sonicated *P. gingivalis* aggravates NAFLD, increases insulin resistance, and inhibits glucose metabolism. Moreover, endotoxemia modifies the gut microbiota (Figure 15). This is the first study to comprehensively assess gene expression profiles in the liver and gut microbiota composition following endotoxemia from *P. gingivalis* injection in mice. In addition to subgingival scaling, tooth brushing poses a risk for bacteremia, similar to dental extraction, in subjects with poor oral health (Lockhart et al., 2008). Intensive periodontal treatment of subjects with poor oral health resulted in acute, short-term, systemic inflammation and endothelial dysfunction (Tonetti et al., 2007). Thus, our results emphasize that maintaining excellent oral health is important for systemic heath.

## Author Contributions

NS performed most of the experiments and wrote the first draft of the manuscript. RK, KW, SM, TS, SU, YT, AO, and YI assisted in some studies and reviewed the manuscript. TK, NM, TH, and MT provided expertise on microarray analysis. TS, YT, and HT provided advice on 16S rRNA sequencing analysis. SK supervised all the studies and the writing of the manuscript.

## Conflict of Interest Statement

The authors declare that the research was conducted in the absence of any commercial or financial relationships that could be construed as a potential conflict of interest.

## Footnotes

1 http://weizhongli-lab.org/cd-hit-otu/

2 https://github.com/lh3/seqtk

3 http://www.webgestalt.org

4 http://david.abcc.ncifcrf.gov/

5 http://software.broadinstitute.org/gsea/index.jsp
