# Supplementary material for: Endotoxemia by Porphyromonas gingivalis Injection Aggravates Non-alcoholic Fatty Liver Disease, Disrupts Glucose/Lipid Metabolism, and Alters Gut Microbiota in Mice
Source: Front Microbiol. 2018 Oct 24;9:2470. doi: 10.3389/fmicb.2018.02470 (PMC6207869; doi:10.3389/fmicb.2018.02470)
Supplement: Supplementary file 1 [file Table_1.DOCX]

**Supplementary information 1**: Animal experimental protocol

A total of 20 male C57BL/6J mice (8-weeks-old; Sankyo Laboratory, Tokyo, Japan) were used in this study, and allowed free access to water and food throughout the experimental period. The mice were fed High-fat Diet 32 (CLEA Japan, Inc., Tokyo, Japan), which contains 506.8 kcal/100 g (57.5% from fat, 19.7% from protein, and 22.8% from carbohydrate). High-fat diet 32 is composed of 24.5% milk casein, 5.0% albumen powder, 0.43% L-cystine, 15.88% powdered beef tallow, 20.0% safflower oil, 5.5% crystalline cellulose, 8.25% Maltodextrin, 6.928% lactose, 6.75% sucrose, 1.4% American Institute of Nutrition (AIN) - 93vitamin mix, 5.0% AIN G mineral mix, 0.36% Choline Hydrogen, 0.002% tertiary butyl hydroquinone. The mice were randomly divided into a control group (HFco, n=10) and a group receiving intravenous injection of sonicated *P. gingivalis* twice a week (HFPg, n=10) for 12 weeks.

A total of 10^8^ colony-forming units (CFU) of sonicated *P. gingivalis* suspended in 100 μl of physiological saline solution was given to the HFPg mice by intravenous injection. HFco mice were given saline only. The mice placed restraining device, mice tail was put into water bath to make it swollen. After 2-3 min, the 29G needle (Terumo Corporation, Tokyo, Japan) was inserted to the expanded vein and injected sonicated *P. gingivalis* or saline.

An oral glucose tolerance test (GTT, n=9) and insulin tolerance test (ITT, n=9) were performed after 6 hours fasting. Glucose 1 g/ kg body weight was gavaged with oral sonde (Fuchigami instrument Corporation, Kyoto, Japan). The blood samples were collected from the tail and blood glucose level was measured at 0, 15, 30, 60, and 120 min for GTT. Insulin 1.5 U/ kg body weight was injected intraperitoneally with 21G needle (Terumo Corporation, Tokyo, Japan) and blood glucose level was measured in a same way for GTT.

Micro- computed tomography (CT) imaging was performed with a RmCT2 micro-CT unit (Rigaku Corporation, Tokyo, Japan) under anesthetised with 2% isoflurane (Wako Pure Chemical Industries, Osaka, Japan).
