# Supplementary material for: Endotoxemia by Porphyromonas gingivalis Injection Aggravates Non-alcoholic Fatty Liver Disease, Disrupts Glucose/Lipid Metabolism, and Alters Gut Microbiota in Mice
Source: Front Microbiol. 2018 Oct 24;9:2470. doi: 10.3389/fmicb.2018.02470 (PMC6207869; doi:10.3389/fmicb.2018.02470)
Supplement: Supplementary file 2 [file Table_2.DOCX]

**Supplementary Table S2**: Primers used for quantitative PCR analysis

| Gene | Primers | |
| --- | --- | --- |
|  | Sense (5'-3') | Anti-sense (5'-3') |
| *Glut2* | GCACAGACACCCCACTTACA | GCAAAGCTGGACACAGACA |
| *G6p* | TGGCCTGGCTTATTGTACCT | GTGCTAAGAGGAAGACCCGA |
| *Glck* | TATGAAGACCGCCAATGTGA | TTTCCGCCAATGATCTTTTC |
| *Pepck* | CAGCCAGTGCCCCATTATT | CCACCAAAGATGATACCCTCA |
| *Srebp1c* | GGAGCCATGGATTGCACATT | GCTTCCAGAGAGGAGCCCAG |
| *Acc1* | ACACCATGTTGGGAGTTGTG | GCTGTTCCTCAGGCTCACAT |
| *Tnfα* | ACGGCATGGATCTCAAAGAC | AGATAGCAAATCGGCTGACG |
| *Il6* | TAGTCCTTCCTACCCCAATTTCC | TTGGTCCTTAGCCACTCCTTC |
| *Tgfb* | GGATACCAACTATTGCTTCAGCTCC | AGGCTCCAAATATAGGGGCAGGGTC |
| *Acot1* | ATTCAAGGGCTGGGAATGGA | TTCTCGCAGCTGGATTGAAC |
| *Acot2* | AAGTGCTGGGAGTAAAGCCA | TTACGGCACTGGGGAATGAA |
| *Acot3* | AAGCTGTGACCTACCTGCTC | TGATGACAGCGGCTGTGATA |
| *Acot4* | GCGGTACATGCTTCGACATC | TGGAAACTGTGGCTGAGACA |
| *Aldh3a2* | AAATCTTAGCAGCCATCGCG | CTCGCAGAAGCCAATTCAGG |
| *Cpt1b* | TTCCTGTACCAACGAGTCCG | TGGCTAGGCGGTACATGTTT |
| *Cyp4a10* | TTGACCCTTCCAGGTTTGCA | GCCACAATCACCTTCAGCTC |
| *Cyp4a14* | AAGACCCTCCAGCATTTCCC | TGTAAGCAGGCACTTGGGAA |
| *Cyp4a31* | AAGGTGTCCAGGTCACACTC | AGGAATGAGTGGCTGTGTCG |
| *Ehhadh* | CCCTCAGGAGCATCTTGGAA | TACAGCAACCACAGGGATCC |
| *36b4* | GCTCCAAGCAGATGCAGCA | CCGGATGTGAGGCAGCAG |
